# Clinical significance of serum triglyceride elevation at early stage of acute biliary pancreatitis

**DOI:** 10.1186/s12876-015-0254-x

**Published:** 2015-02-14

**Authors:** Long Cheng, Zhulin Luo, Ke Xiang, Jiandong Ren, Zhu Huang, Lijun Tang, Fuzhou Tian

**Affiliations:** 1 Department of General Surgery, Chengdu Military General Hospital, Jinniu District, Chengdu, Sichuan Province PR China 610083; 2 Department of Pharmacy, Chengdu Military General Hospital, Chengdu, Sichuan Province People’s Republic of China

**Keywords:** Acute pancreatitis, Biliary pancreatitis, Hypertriglyceridemia, Triglyceride

## Abstract

**Background:**

Pancreatitis induced by hypertriglyceridemia (HTG) has gained much attention. However, very limited numbers of studies have focused on the clinical significance of TG elevation in non-HTG induced pancreatitis, such as acute biliary pancreatitis (ABP). This study aimed to study the clinical significances of triglyceride (TG) elevation in patients with ABP.

**Methods:**

We retrospectively analyzed a total of 426 ABP cases in our research center. According to the highest TG level within 72 h of disease onset, the patients were divided into a normal TG group and an elevated TG group. We analyzed the differences between the two groups of patients in aspects such as general information, disease severity, APACHE II (acute physiology and chronic health evaluation II) and Ranson scores, inflammatory cytokines, complications and prognosis.

**Results:**

Compared with the normal TG group, patients in the elevated TG group showed a significantly higher body mass index and were significantly younger. TG elevation at the early stage of ABP was associated with higher risk of severe pancreatitis and organ failures, especially respiratory failure. For patients with severe pancreatitis, those with elevated TG levels were more likely to have a larger area of necrosis, and higher incidence of pancreatic abscess as well as higher mortality (17.78% versus 9.80%, P < 0.05).

**Conclusions:**

In ABP patients, TG elevation might participate in the aggravation of pancreatitis and the occurrence of systemic or local complications. Thus, the TG level may serve as an important indicator to determine the prognosis of patients with ABP.

## Background

Acute pancreatitis (AP) is a life-threatening inflammatory disease involving the pancreas as well as peripancreatic and even distant organs [1]. The main causes of AP include biliary tract disease, alcohol abuse, congenital anomaly, drugs, etc. [2]. In recent years, the relationship between hypertriglyceridemia (HTG) and AP has received widespread attention from scholars, and it is generally believed that blood triglyceride (TG) levels greater than 11.3 mmol/L can directly induce AP [3,4]. However, HTG-induced AP is uncommon, accounting for only 1-4% of AP cases. On the other hand, HTG is commonly present at the early stage of non-HTG-induced AP, such as acute biliary pancreatitis (ABP), and its clinical significance remains unclear.

It has been reported that more than 50% of non-HTG-induced AP patients demonstrate mild-to-moderate TG elevation [5]. However, the relationship between the elevated TG level and severity of non-HTG-induced AP is not well documented. Some studies reported that when non-HTG-induced AP was accompanied by TG elevation, the disease course of AP shows a trend for aggravation; in particular, if the serum TG level is reduced under 5.65 mmol/L, the disease condition will be gradually stabilized and improved [6-8]. However, some other studies showed that an increased TG level might just represent a symptom associated with AP, and there is no significant relationship between an elevated TG level and the severity and prognosis of AP patients [9]. This discrepancy may be due to the fact that the AP cases included in these studies were induced by different causes, and HTG might play different roles in AP cases induced by different causes.

Therefore, investigating the clinical significance of an elevated TG level in AP induced by a single homogeneous cause could more accurately illustrate the effects of mild-to-moderate HTG on the severity and prognosis of AP patients. As acute biliary pancreatitis (ABP) remains the main type of AP in China and many other countries, the current study focused on the clinical significance of HTG in ABP patients.

## Methods

### Patients

#### Patient selection

This study retrospectively analyzed 426 ABP cases that were treated in our hospital. The diagnosis and classification of the severity of AP were performed according to the 2012 revision of the Atlanta Classification [10]. The diagnosis of ABP was based on the standards proposed in the literature with the following minor amendments [11]: 1) confirmed diagnosis of AP; 2) no history of hyperlipidemia or alcohol abuse; 3) abdominal ultrasonography (AUS), computed tomography (CT), magnetic resonance cholangiopancreatography (MRCP), and endoscopic ultrasound (EUS) detection of gallstones or biliary sludge, or laboratory tests showing two of the following items: ALT >75U/L, ALP >125 U/L, and TBIL >2.3 mg/dl. This study was performed according to the principles of the Declaration of Helsinki (modified 2000) and was approved by the ethics committee of Chengdu Military General Hospital (No. 2013037).

#### Treatment protocols

At the outset, all patients were treated in a conservative manner, with fluid resuscitation and antibiotics, as described in the literature [12,13]. Endoscopic retrograde cholangiopancreatography (ERCP) and endoscopic sphincterotomy (EST) were performed in patients with jaundice and cholangitis. Percutaneous catheter drainage (PCD) were carried out to eliminate the debris and collections in the (peri)pancreas, similar to other reports [14]. The number and size of the catheters were determined by the size, viscosity, and location of the debris and collections. In accordance with widely accepted consensus [15], if there was no clinical improvement or if ongoing necrosis with bowel complications was present, open necrosectomy with closed lesser-sac drainage and postoperative lavage was performed.

### Methods

#### Group division

According to the highest TG level within 72 h of disease onset, the patients were divided into a normal TG group (<1.88 mmol/L) and an elevated TG group (≥1.88 mmol/L).

#### Data collection

In addition to the collection of general information including sex, age, body mass index (index), etc., we specially analyzed differences between the normal TG group and the elevated TG group in aspects such as pancreatitis severity score, incidence of systemic complications, incidence of local complications, mortality, days in hospital and intensive care unit and care cost. Differences in levels of inflammatory cytokines within 72 h of disease onset—C-reaction protein (CRP), interleukin 6 (IL-6), IL-10, tumor necrosis factor-alpha (TNF-α) —between the two groups were also analyzed. Initial contrast-enhanced computed tomography was performed within the first week of the onset of disease and repeated depending on the indication. The images obtained were double-checked by two experienced radiologists. Meanwhile, extent of necrosis were measured and classified into three grades, <30%, 30-50% and >50%, according to Balthazar classification. Acute physiology and chronic health evaluation II (APACHE II) score and CTSI were calculated for each patient at the time of admission and serially calculated before and after each type of intervention.

#### Definition and criteria

The classification of AP severity was based on the 2012 revision of the Atlanta Classification, and the Ranson score and APACHE II (acute physiology and chronic health evaluation II) score were calculated within 24 ~ 48 h after patient admission.

The diagnostic criteria for systemic complications of AP were as follows: shock, with systolic blood pressure ≤80 mmHg and a duration of 15 min; respiratory failure, with PaO2 ≤ 60 mmHg; renal failure, with serum creatinine >176.8 μmol/L; coagulation disorders, with prothrombin time (PT) less than 70% that of normal people and/or partial thromboplastin time (PTT) > 45 s; sepsis, with a body temperature (T) > 38.5°C, white blood cell (WBC) count >6.0 × 10^9^/L, base excess ≤ 4 μmol/L, a duration of 48 h, and positive blood/ aspirate bacterial culture; systemic response syndrome, with T >38.5°C or <36°C, WBC count >12.0 × 10^9^/L or <4.0 × 10^9^/L, pulse >90 beats/min, and respiratory rate >20 breaths/min or PCO2 < 32 mmHg; upper gastrointestinal bleeding; and pancreatic encephalopathy. Local complications of AP included pancreatic necrosis, inanimate pancreatic tissue, or peripancreatic fat as revealed by enhanced CT and pancreatic pseudocyst, with liquid accumulation wrapped in a complete and non-epithelial envelope and enclosed pancreatic secretions, granulation tissue, and fibrous tissue.

#### Statistical methods

The statistical analysis was carried out using SPSS version 16.0 for Windows (SPSS Inc., Chicago, IL). Normality of data was determined by Kolmogorov–Smirnov tests of normality. Data were expressed as mean ± standard deviation for normally distributed data and median and interquartile range for non-normally distributed data. For normally distributed data, variables were compared using Student’s t test for two groups. For skewed data, the Mann–Whitney test was used. Qualitative or categorical variables were described as frequencies and proportions. Proportional variables were compared using the χ2 test or Fisher’s exact test. All statistical tests were two-tailed and performed at a significance level of P < 0.05.

## Results

### Comparison of general information of patients

There were 426 ABP cases included, of which 289 (67.84%) were assigned to the normal TG group and 137 (32.16%) to the elevated TG group. The mean levels of TG in the two groups were 1.21 mmol/L and 6.52 mmol/L, respectively. This cohort included 188 male and 238 female patients, and the male/female ratio of the elevated TG group was significantly higher than that of the normal TG group (72/65 versus 116/173, p < 0.05). In addition, the age of the patients in the elevated TG group was significantly lower than that in the normal TG group (46.35 ± 7.26 versus 54.89 ± 7.84, p < 0.05), while the body mass index (BMI) of the elevated TG group was significantly higher than that of the normal TG group (28.35 ± 5.13 versus 22.57 ± 4.42, p < 0.01), Table 1. Meanwhile, the frequencies of manifestation of cholangitis between the two groups were not significantly different, 22.15% (64/289) versus 23.36% (32/137). These results showed that younger male ABP patients with a higher BMI were more likely to be complicated with TG elevation.

**Table 1:** **The characteristics of ABP patients enrolled in this study**

characteristic	Normal TG group	Elevated TG group	P value
**Number of patients**	289	137	
**Demographic data**			
Age (mean ± SD)	54.89 ± 7.84	46.35 ± 7.26	0.011*
Male/female	116/173	72/65	0.027*
BMI	22.57 ± 4.42	28.35 ± 5.13	0.001*
Referral after onset of symptoms (hours) (mean ± SD)	26.8 ± 16.8	24.5 ± 15.2	0.402
**TG level (mmol/L)**	1.21 ± 0.56	6.52 ± 1.52	0.001*
**Manifestation of cholangitis (%)**	22.15% (64/289)	23.36% (32/137)	0.435
**Medical economics (median ± interquartile range)**			
Days in hospital	34.7 ± 16.42	39.5 ± 18.54	0.375
Days in intensive care unit (ICU)	5.2 ± 2.36	6.4 ± 3.46	0.562
Total cost during hospitalization (Dollars)	5646.1 ± 1432.32	6103.2 ± 2140.29	0.781

TG, triglyceride; BMI, body mass index. *Significant difference.

### Relationship between TG level and ABP severity

The total incidence rate of moderately severe acute pancreatitis (MSAP) and severe acute pancreatitis (SAP) in this study group was 22.54% (96/426); in particular, SAP and MSAP patients accounted for 32.85% (45/137) of the cases in elevated TG group, and this rate was significantly higher than that in the normal TG group (p < 0.05). Laboratory detection showed that the CRP level in elevated TG group was remarkably higher than that in normal TG group (p < 0.05). Meanwhile, patients in the elevated TG group had APACHE II scores and Ranson scores that were significantly higher than those of patients in the normal TG group (p values were <0.01 and <0.05, respectively), Table 2. Correlation analysis showed that TG levels and APACHE II scores were significantly correlated (p < 0.05, R = 0.665) in patients in the elevated TG group, whereas TG levels were not significantly correlated with the Ranson score. These results suggest that elevated TG levels in the early phase of ABP may be involved in the progress of the disease.

**Table 2:** **The Laboratory and clinical parameters between two groups**

Items	Normal TG group	Elevated TG group	P value
**Number of patients**	289	137	
**Severity classification**			
MAP	238 (82.35%)	94 (68.61%)	0.157
MSAP	32 (11.07%)	28 (20.44%)	0.021*
SAP	19 (6.57%)	17 (12.41%)	0.015*
**Laboratory parameters**			
C.reaction protein (CRP) (mg/L)	36.2 ± 15.33	61.2 ± 18.41	0.038*
IL-6 (pg/L)	54.5 ± 20.41	62.6 ± 22.42	0.241
IL-10 (pg/L)	30.6 ± 17.48	35.1 ± 18.59	0.672
TNF-α (pg/L)	8.2 ± 3.53	9.8 ± 3.77	0.521
**Severity scores**			
APACHIIscore (mean ± SD)	6.26 ± 3.48	10.11 ± 3.62	0.007*
Ranson score (mean ± SD)	1.68 ± 0.79	2.34 ± 0.93	0.042*
Marshall score (mean ± SD)	2.1 ± 0.57	2.6 ± 0.68	0.142

TG, triglyceride; MAP, mild acute pancreatitis; MSAP, moderately severe acute pancreatitis; SAP, severe acute pancreatitis; APACHE II, acute physiology and chronic health evaluation II. *Significant difference.

### Involvement of TG elevation in the occurrence of systemic complications in severe ABP patients

With regard to the severe cases (including MSAP and SAP), the incidence of systemic organ failure in elevated TG group (33.33%, 17/51) was significantly higher than that in the normal TG group (46.67%, 21/45), p < 0.05), Table 3. Statistical analysis showed that severe ABP patients with elevated TG levels had a significantly increased risk of organ failures involving single organ failure. Among patients with moderate-severe degrees of severe pancreatitis, the incidence of respiratory failure was significantly higher in the elevated TG group than the normal TG group (28.89% (13/45) versus 13.73% (7/51), p < 0.05). However, these two groups did not show significant differences in the incidence rates of renal failure, circulatory failure, coagulation disorders, and other types of organ failure (p > 0.05). These results suggest that ABP patients with elevated TG levels are more likely to develop organ failure, particularly respiratory failure.

**Table 3:** **Incidence of organ failure in MSAP or SAP patients**

Items	Normal TG group	Elevated TG group	P value
**Number of patients**	51	45	
**Total organ failure**	17 (33.33%)	21 (46.67%)	0.036*
Single	11 (21.57%)	15 (33.33%)	0.047*
Multiple	6 (11.76%)	6 (13.33%)	0.761
**Respiration**	7 (13.73%)	13 (28.89%)	0.022*
**Circulation**	5 (9.80%)	3 (6.67%)	0.535
**Kidney**	6 (11.76%)	5 (11.11%)	0.882
**Coagulation**	4 (7.84%)	3 (6.67%)	0.682
**Other organ or system**	4 (7.84%)	3 (6.67%)	0.682

TG, triglyceride; MSAP, moderately severe acute pancreatitis; SAP, severe acute pancreatitis. *Significant difference.

### Effect of TG elevation on the development of local complications in patients with ABP

Among patients with moderate-severe degrees of severe ABP, 55.56% of patients with elevated TG levels had pancreatic necrosis, and 50.98% of patients with normal TG had pancreatic necrosis, with no significant difference between these two groups. However, patients with elevated TG levels showed significantly higher probabilities of developing necrosis over a relatively large area (with a necrotic area greater than 30%) and a higher incidence of pancreatic abscess compared to patients with normal TG levels, Table 4. For patients with moderate-severe degrees of severe ABP, there was no significant difference in the incidence rates of pseudocysts between patients with elevated TG and patients with normal TG levels. These results suggest that although TG elevation did not increase the total incidence of pancreatic necrosis in ABP patients, TG elevation may increase the scope and extent of pancreatic necrosis once necrosis occurs.

**Table 4:** **Occurrence of local complications in MSAP or SAP patients**

Items	Normal TG group	Elevated TG group	P value
**Number of patients**	51	45	
**Incidence of necrosis**	34 (66.67%)	36 (80.00%)	0.253
**Maximum extent of necrosis**			
Less than 30%	18 (35.29%)	12 (26.67 %)	0.137
30% ~ 50%	10 (19.61 %)	15 (33.33%)	0.027*
More than 50%	6 (11.76 %)	9 (20.00%)	0.002*
**Pancreatic abscess**	4 (7.84 %)	8 (17.78%)	0.001*
**Pseudocyst**	6 (11.76 %)	5 (11.11%)	0.683

*Significant difference.

### Association of TG level with treatments and prognosis of ABP patients

All mild ABP patients were treated in a conservative manner and were discharged after rehabilitation. There were 41.87 % of mild ABP patients (139/332) underwent ERCP/EST, and no significant difference was observed between normal TG group and elevated TG group. Besides ERCP/EST,PCD and open necrosectomy were also performed in some of patients with moderate or severe ABP. For patients with MSAP/SAP, although the frequencies of ERCP/EST were not significantly different between normal TG group and elevated TG group (47.06% (24/51) versus 46.67% (21/45)), more patients in elevated TG group underwent PCD and open necrosectomy, Table 5. The mortality rate of severe ABP patients with elevated TG was 17.78% (8/45), whereas that of severe ABP patients with normal serum TG was 9.80% (5/51); the difference between the two groups was significant (p < 0.05). The patients were followed for 1 year, and we found no significant differences in the recurrence rate and life quality of patients after discharge. These results showed that TG elevation may be a manifestation indicating poor prognosis in patients with ABP.

**Table 5:** **Treatment and prognosis for MSAP or SAP patients**

Items	Normal TG group	Elevated TG group	P value
Number of patients	51	45	
ERCP or EST	24 (47.06%)	21 (46.67%)	0.566
PCD	20 (39.12%)	27 (60.00%)	0.033*
Open necrosectomy	3 (5.88%)	9 (13.33%)	0.037*
mortality	5 (9.80%)	8 (17.78%)	0.011*
recurrence rate within 1 year	10 (19.61%)	11 (24.44 %)	0.372

ERCP, Endoscopic retrograde cholangiopancreatography; and EST, endoscopic sphincterotomy; PCD, Percutaneous catheter drainage. *Significant difference.

## Discussion

In recent years, the relationship between HTG and AP has received widespread attention from scholars, and it is generally believed that a serum TG level >11.3 mmol/L can directly induce AP. However, despite the increasing incidence of AP induced by HTG, this type of AP is not the major type observed in clinical practice. The more common clinical situation is that non-HTG-induced AP patients were complicated with TG elevation, and it has been reported in the literature that approximately 50% of non-HTG-induced AP patients demonstrate mild-moderate TG elevation. However, there have been only limited numbers of studies on the significance of TG elevation in non-HTG induced pancreatitis, such as acute biliary pancreatitis (ABP), and the results were inconsistent.

It also remains controversial whether TG elevation is an associated etiological factor or a concomitant symptom in non-HTG induced AP patients. In alcoholic pancreatitis, alcohol can directly affect the metabolism of TG, causing elevated TG levels, and is therefore directly involved in the occurrence of pancreatitis. Thus, an elevated TG level can be seen as an associated etiological factor of alcoholic pancreatitis [16], whereas it may be considered as concurrent symptom of biliary pancreatitis [17]. In addition, the relationships between TG elevation and the severity and prognosis of AP, as reported in the literature, are also inconsistent. For example, studies have reported that when AP induced by non-HTG causes is complicated with TG elevation, the disease course of AP shows a trend for aggravation [7,8]. However, other studies show that an increased TG level may only represent a symptom associated with AP, as there is no significant relationship between TG level and the severity and prognosis of AP patients [9]. The presence of these controversies is likely due to the analysis of different compositions of AP patient, including patients with biliary AP, alcoholic AP, cryptogenic AP, and AP due to other factors, as HTG is known to play different roles in AP caused by different factors.

Therefore, the study of the clinical significance of an elevated TG level in AP due to a single etiological factor could accurately illustrate the effects of mild-moderate HTG on AP disease condition and prognosis. Because biliary tract disease is a major cause of AP in China and other Eastern countries, we chose to study the effects of TG elevation on the disease condition and prognosis of patients with ABP. When selecting cases, we excluded patients with a history of hyperlipidemia and alcoholism, aiming to exclude cases with elevated TG levels before the onset of pancreatitis. During the occurrence of AP, due to the body’s stress response, serum catecholamine and glucagon levels, as well as lipase activity, are increased, leading to accelerated break down of fat tissue and the subsequent release of TG and increase in serum lipid concentrations [18,19]. In this study group, the incidence of SAP in ABP patients associated with elevated TG was significantly higher compared to that in patients without elevated TG; in addition, ABP patients with elevated TG levels also showed more severe pancreatic necrosis. These results may be due to the fact that elevated TG might lead to increased blood viscosity, which further promotes blood circulation disorders of the pancreas. In addition, large quantities of free fatty acids released from TG breakdown damage the pancreatic acinar cells and capillaries, leading to a higher likelihood of SAP [20,21]. Furthermore, this result might also be related to the participation of cholecystokinin, as some studies have shown that in the presence of TG elevation, free fatty acids produced in the body can strengthen the stimulation of cholecystokinin on the excretion function of pancreatic acinar cells, which subsequently activates the endoplasmic reticulum stress phenomenon and thereby damages pancreatic acinar cells [22].

This study also found that ABP patients with elevated TG levels were more prone to organ failure, particularly respiratory failure. In the AP disease course, respiratory complications primarily include hypoxemia, acute respiratory distress syndrome, atelectasis, and pleural effusion. Respiratory failure is largely due to the large number of toxic cytokines produced during the AP disease course, mainly including platelet-activating factor, TNF-α, IL-1, IL-6, IL-8, NO, P substance, and macrophage-secreted cytokines, which can cause systemic inflammatory response syndrome and respiratory dysfunction. The reason why ABP patients with elevated TG levels were more likely to experience respiratory failure is related to the functions of free fatty acids. In the presence of elevated TG, serum TG is broken down under the action of lipoprotein lipase in the lung, leading to the production of large quantities of free fatty acids. Subsequently, free fatty acids dissociate from albumin in the blood, enter the alveolar capillary membrane, and destruct the pulmonary blood microcirculation, leading to respiratory failure [23,24].

It remains unclear why some ABP patients show elevated TG levels while others have normal TG levels, and why the degree of elevated TG varies among patients. We believe that this difference may be related to a number of factors. First, the local or whole body fat distribution of patients may be associated with elevated TG levels and the degree of such elevation. When the body has high levels of fat, under the same level of stress and level of lipase activity, the amount of involved fat tissue is increased, and the amount of TG release is increased. In the present study, the BMI of ABP patients with elevated TG levels was significantly higher than that in patients with normal TG levels. Indeed, previous studies have shown that obesity is closely associated with AP aggravation, and some scholars have suggested that obesity should be considered an independent risk factor of AP aggravation, which is consistent with the results of this study. In addition, in recent years, some scholars have found that genetic polymorphisms are associated with the pathogenesis of AP with HTG [25]. For example, Chang and his colleagues [26] first reported that in HTG-induced AP patients, lipoprotein lipase gene mutation was significantly increased, and the probability of AP was 77.8% in patients with hyperlipidemia caused by the mutation in this gene. In short, there may be a mutually reinforcing cycle between elevated TG levels and pancreatitis, such that the occurrence of pancreatitis leads to elevated TG levels, which further increase the severity of pancreatitis. This cycle may represent another important vicious cycles in AP pathogenesis, and timely blockade of this cycle may hold important significance for reducing the degree of AP aggravation and improving the efficacy and prognosis for patients with SAP.

The general treatment protocol of ABP was similar to AP induced by other causes. At the outset, all patients were treated in a conservative manner, and subsequently so-called step-up approach was carried out [27,28]. Meanwhile, ERCP and EST were selectively performed in some ABP patients, especially for patients with jaundice and cholangitis. However, there were still controversies on the indications and outcomes of ERCP and EST in ABP treatment [29,30]. In the current study, no significant difference of the frequencies of ERCP/EST was observed between normal TG group and elevated TG group. For patients with MSAP/SAP, the frequencies of ERCP/EST were also not significantly different between normal TG group and elevated TG group, but more patients in elevated TG group underwent PCD and open necrosectomy. These results indicated that the ABP patients with TG elevation might be liable to undergo PCD and open necrosectomy. However, as the patients were divided into two groups according to the highest TG level within 72 h of disease onset, we presumed that the raised TG level might be responsible for the liability of PCD and open necrosectomy.

## Conclusions

In summary, when ABP is accompanied with TG elevation, the risks of SAP and organ failure (especially respiratory failure) are significantly higher compared to patients with normal TG levels. In particular, elevated TG levels and pancreatitis may form a mutually reinforcing cycle, and timely implementations of measures to block this vicious cycle may hold important significance for reducing the rate of AP aggravation and improving the treatment effects and prognosis of SAP patients. Nevertheless, further research is needed to reveal the pathophysiological mechanisms for why elevated TG levels are present in ABP patients, as well as the mechanism by which elevated TG cause injury to specific tissues and organs.

## Abbreviations

AP: Acute pancreatitis
TG: Triglyceride
ABP: Acute biliary pancreatitis
HTG: Hypertriglyceridemia
APACHE II: Acute physiology and chronic health evaluation II
AUS: Abdominal ultrasonography
CT: Computed tomography
MRCP: Magnetic resonance cholangiopancreatography
EUS: Endoscopic ultrasound
